# Pleomorphic adenoma: a rare presentation in buccal salivary gland with extensive squamous and mucous metaplasia

**DOI:** 10.11604/pamj.2019.33.147.17550

**Published:** 2019-06-26

**Authors:** Aadithya Basavaraj Urs, Jeyaseelan Augustine, Deepika Negi, Rudra Deo Kumar, Sujoy Ghosh

**Affiliations:** 1 Department of Oral Pathology and Microbiology, Maulana Azad Institute of Dental Sciences, New Delhi, India; 2 Department of Oral and Maxillofacial Surgery, Maulana Azad Institute of Dental Sciences, New Delhi, India; 3 Department of Oral Medicine and Radiology, Maulana Azad Institute of Dental Sciences, New Delhi, India

**Keywords:** Buccal mucosa, mucous metaplasia, pleomorphic adenoma, squamous metaplasia

## Abstract

Pleomorphic Adenoma (PA) is the most common salivary gland tumor and accounts for about 60% of all salivary gland neoplasms. Intraorally, the hard palate is the most common presenting site (50-60%) followed by upper lip (15-20%) and rarely buccal mucosa (8-10%). Histopathologically, PA shows diverse morphology resulting from amalgamation of cellular and stromal components. The PA may show changes in the stromal and epithelial components, such as sebaceous, lipocytic and oncocytic metaplasia. A rare characteristic of PA is to show extensive squamous and mucous differentiation which poses diagnostic dilemma to the pathologist. Here, we present an unusual case of PA of buccal minor salivary gland with squamous and mucous metaplasia. The localization, gender and microscopic features of the presented case are unusual.

## Introduction

Pleomorphic adenoma (PA) is a benign salivary gland tumor. The “pleomorphic” nature of the tumor can be explained on the basis of its epithelial and connective tissue elements [1]. The incidence of PA in intraoral minor salivary glands is approximately 40-50% and presents with a slight female predilection. It occurs over a wide age range, but the mean age is 43.6 years and the peak incidence is between the fourth and fifth decades of life [2, 3].

## Patient and observation

**Figure 1:**
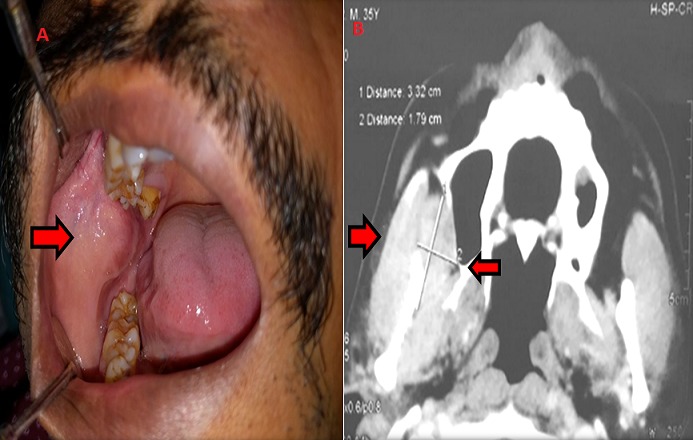
A) intraoral photograph showing well defined solitary swelling in right buccal mucosa; B) CECT showing heterogeneously enhancing lobulated soft tissue lesion in the right masticator space between masseter and pterygoid muscle (arrows)

A 35 year old male patient reported to the outpatient department (OPD) of Maulana Azad Institute of Dental Sciences with a chief complaint of painless swelling over the right side of face since 4 years. The swelling was preceded by extraction in the same region 4 years back. The swelling was initially small in size; gradual increase to present size was noticed by the patient since 2 months. Patients also complained of recurrent cheek biting in the same area, however, there was no history of pus or blood discharge from the swelling. The patient had a history of betel quid (areca nut, slaked lime and tobacco) chewing 2 to 3 times per day since 2 years and had quit the habit 5 months back. On general and systemic examinations, the patient reported with renal stone and was under medication for the same since 2 years. There was no regional lymphadenopathy. On extraoral examination, facial asymmetry was observed. A solitary dome shaped, oval swelling with smooth surface was present on right cheek region with normal overlying skin. Swelling was approximately 4 × 3cm in size and extending from 3cm below infraorbital margin to 2cm above the inferior border of mandible superio-inferiorly. Antero-posteriorly, it extended 2cm from right ala of nose to 3cm anterior to angle of mandible. Mouth opening was adequate. On intraoral examination, the swelling was soft to firm in consistency, mobile, non-fluctuant, non-pulsatile, non-tender, and was mobile in all planes (Figure 1 A). The color of the swelling was same as that of adjacent mucosa. Local temperature over the swelling was not raised. Ultrasonography (USG) from the lesion revealed well-defined, heteroechoic solid lesion measuring 2.0 X 2.0 X 2.7cm in size on right cheek deep to masseter muscle and anterior to the ramus of mandible. The lesion showed little peripheral arterial vascularity.

**Figure 2:**
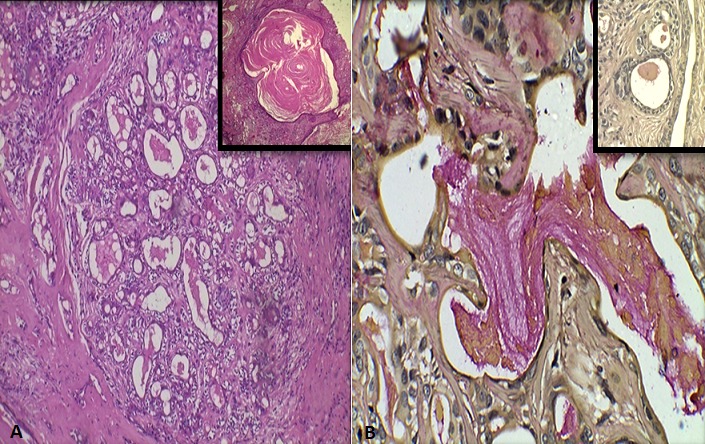
A) photomicrograph showing ductal and myoepithelial in a mesenchymal background (H&E X10). Inset shows squamous metaplasia (H&E X40); B) positive mucicarmine staining indicating mucous metaplasia within the ductal cells, (mucicarmine X40)

**Figure 3:**
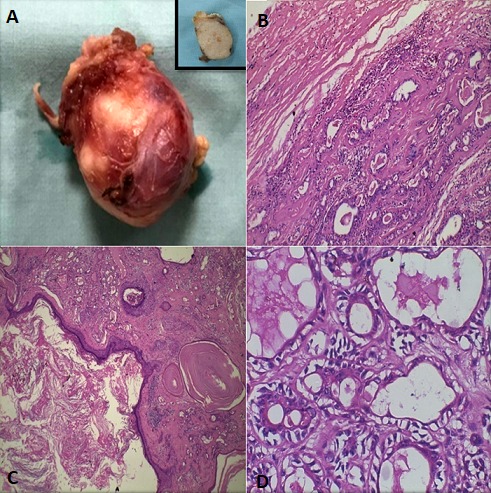
A) gross examination of the tissue shows well encapsulated, ovoid mass of size approximately 2.5 x 2.5 x 3cm. Cut surface shows whitish solid area (inset) intermixed with yellow areas; B) Well encapsulated lesion showing thick fibrous capsule and myoepithelial cells in a chondromyxoid background (H&E X10); C) Abundant cystic-squamous lined structures filled with keratin (H&E X40); D) Double layered ductal structures characteristic of PA (H&E X40)

Computed tomography (CT) scan of maxilla and mandible revealed heterogeneously enhancing, lobulated soft tissue lesion, measuring 3.3 X 1.78cm in the right masticator space between masseter and pterygoid muscle causing obliteration of retro-maxillary fat (Figure 1 B). The lesion was seen to indent the right buccal mucosa anteriorly. However, no irregularity of buccal mucosa was seen. The lesion abutted the postero-lateral wall of right maxillary sinus. There was no evidence of associated bony erosions. On clinical and radiographic examination a provisional diagnosis of fibroma was considered. Lipoma, traumatic neuroma, schwannoma and squamous cell carcinoma were kept in the differential diagnosis. Routine hematological investigations were within normal limits. Incisional biopsy was performed and histopathological examination revealed lesional tissue composed of ductal and myoepithelial cells with clear cell changes. At areas keratin pearl formation was seen (Figure 2 A). Mucous metaplasia was also observed at many areas which was confirmed by positive mucicarmine staining (Figure 2 B). Therefore, a final diagnosis of PA with mucous metaplasia was given. Under local anaesthesia, a mucosal incision was given over the mass surface, 1cm away from the Stenson's duct orifice. Blunt and sharp dissection was done alternately to expose the encapsulated mass which was attached to the opening of stensen's duct. The mass was excised completely with adequate margin of normal tissue. The Stenson's duct opening was cannulated and opening of the duct sutured to the mucosal incision. On macroscopic examination, tissue was well encapsulated, ovoid in shape, light brown in color and firm in consistency. Cut surface of the tissue showed whitish solid area with many places showing yellowish color (Figure 3 A). The histopathological examination of excisional tissue showed a covering of thick fibrous capsule (Figure 3 B) underneath which ductal epithelial cells was surrounded by myoepithelial cells in a chondromyxoid background. The ductal cells were columnar, cuboidal and flat at areas while the myoepithelial cells showed spindle, cuboidal, stellate, epithelioid and plasmacytoid shapes. Mucin filled cystic spaces were seen at many places (Figure 3 B). Abundant cystic-squamous lined structures filled with keratin were observed indicating squamous differentiation with keratinization (Figure 3 C). Double layered ductal structures were also observed (Figure 3 D). This confirmed the diagnosis of PA with extensive squamous and mucinous metaplasia. The patient is under regular follow-up.

## Discussion

Pleomorphic Adenoma (PA) is a benign salivary gland tumor with wide cytomorphologic architectural diversity. The diagnosis of PA is established on the basis of history, clinical examination, cytology, and histopathology. Other advanced imaging techniques like CT and USG provide information regarding location, size and extension of tumor to surrounding structures. In our case, on clinical examination, a differential diagnosis of fibroma was kept in mind considering the site, presentation and duration of this painless swelling. The possibility of lipoma was also considered in view of the duration, history of recurrent cheek biting and consistency of the swelling. A traumatic neuroma or schwannoma were also considered as differential diagnosis keeping in mind the history of extraction during which trauma to adjacent structures is likely. Squamous cell carcinoma was also considered in light of habit history of the patient. CT and USG revealed the unusual location of the tumor which was deep to the masseter, involving the masticator space. Bone involvement or bony erosion was not evident.

Histopathologically, tumor tissue in PA consists of an inner cell layer of epithelial cells exhibiting the ability to differentiate into ductal and nonductal cells and an outer layer of myoepithelial cells responsible for the characteristic mesenchymal (chondroid, myxoid and osseous) changes. Foci of hyalinization of collagen, bone and even adipose tissue can be noted in the connective tissue stroma of many tumors [4]. However, the proportion of each component varies widely among different cases and even in individual tumors and pose a considerable diagnostic challenge. PA shows some degree of squamous metaplasia in about 25% of cases [5, 6]. Varying degrees of squamous differentiation are evident in many salivary gland tumours like mucoepidermoid carcinoma (MEC), PA, basal cell adenoma and Warthin's tumour. The reason for the metaplastic process to be triggered is ischemia in most cases. Light and electron micrographs show that the principal portion of salivary gland tissue undergoing squamous metaplasia is the acinar-intercalated duct cell complex. Early stages of this process involve a gradual de-differentiation of acinar cells and hyperplasia of acinar, duct luminal cells and myoepithelial cells. Subsequent to this, both luminal and myoepithelial cells start accumulating tonofilaments with formation of desmosomes and the centrally located cells may undergo keratinization. Ultra-structural studies in MEC have also suggested that squamous differentiation can occur in either luminal, epithelial or modified myoepithelial cells [7]. The marked ability of acinar units in rat salivary gland to undergo squamous metaplasia lends further support to this premise [8]. However, the biologic behavior of PA appears to remain independent of the proportion of squamous and mucous differentiation. The surgical treatment for the PA arising from minor salivary glands is complete wide surgical excision with good safety margins. The prognosis is good and the chances of recurrences are rare.

## Conclusion

Pleomorphic Adenoma (PA) involving buccal mucosa especially deep to masseter is a rare lesion. Clinical examination and histopathological study are required for definitive diagnosis and may pose a challenge even to the most experienced oral pathologist. PA with extensive squamous or mucous metaplasia can pose significant diagnostic challenge as it can be easily confused with other epithelial malignancies showing squamous differentiation such as squamous cell carcinoma or malignant salivary gland pathology showing mucous and squamous differentiation like mucoepidermoid carcinoma. Absence of malignant features is the key diagnostic feature in differentiating it from PA.

## Competing interests

The authors declare no competing interests.
